# Prognostic factors for aggressiveness in subcentimeter papillary thyroid carcinoma: impact of tumor size and lymph node metastases

**DOI:** 10.20945/2359-4292-2023-0422

**Published:** 2024-07-30

**Authors:** Yusuf Kayhan, Leyla Azizova, Merve Yılmaz, Muhsine Bakış, Mehmet Kefeli, Elif Kılıç Kan, Ayşegül Atmaca, Ramis Çolak

**Affiliations:** 1 Ondokuz Mayis University Faculty of Medicine Department of Endocrinology and Metabolism Samsun Turkey Ondokuz Mayis University, Faculty of Medicine, Department of Endocrinology and Metabolism, Samsun, Turkey; 2 Ondokuz Mayis University Faculty of Medicine Department of Internal Medicine Samsun Turkey Ondokuz Mayis University, Faculty of Medicine, Department of Internal Medicine, Samsun, Turkey; 3 Samsun State Gazi Hospital Endocrinology and Metabolism Clinic Samsun Turkey Samsun State Gazi Hospital, Endocrinology and Metabolism Clinic, Samsun, Turkey; 4 Ondokuz Mayis University Faculty of Medicine Department of Medical Pathology Samsun Turkey Ondokuz Mayis University, Faculty of Medicine, Department of Medical Pathology, Samsun, Turkey

**Keywords:** Subcentimeter papillary thyroid carcinoma, papillary thyroid microcarcinoma, tumor size, prognosis, lymph node metastasis, surgical pathology

## Abstract

**Objective:**

Subcentimeter papillary thyroid carcinoma (sPTC), also known as papillary thyroid microcarcinoma, is associated with a good prognosis and low mortality risk. However, some sPTCs exhibit biologically aggressive characteristics. The aim of this study was to identify factors affecting the prognosis and aggressiveness of sPTC by considering the demographic characteristics of patients with sPTC and the pathologic characteristics of the tumors.

**Subjects and methods:**

The study included 255 patients aged ≥ 18 years who were operated on at Ondokuz Mayis University, Faculty of Medicine (Samsun, Turkey) between June 2008 and December 2021. All patients had histopathologic confirmation of sPTC (≤10 mm) and underwent regular follow-up for at least 36 months.

**Results:**

The tumors had a mean size of 5 mm (0.1-10 mm) and were multifocal in 53.7% of patients. Capsular invasion was observed in 9% of patients. Vascular invasion, lymphatic invasion, and extrathyroidal invasion were present in 2%, 5.5%, and 0.8% of patients, respectively. Metastatic cervical lymph nodes were observed in 9.4% of patients. On multivariate logistic regression analysis, tumor size (odds ratio [OR] 1.380, 95% confidence interval [CI] 1.106-1.722, p = 0.004) and sex (OR 4.233, 95% CI 1.355-13.226, p = 0.013) were the main predictive factors influencing lymph node metastasis. Tumors > 5 mm, compared with tumors ≤ 5 mm, had higher rates of multifocality (p = 0.009), parenchymal invasion (p = 0.008), calcifications (p = 0.001), microscopic lymphatic invasion (p = 0.002), and presence of metastatic lymph nodes (p < 0.001).

**Conclusion:**

The findings of this study highlight important factors to consider in making decisions about prophylactic central compartment neck dissection in patients with sPTCs, particularly those with clinically node-negative tumors.

## INTRODUCTION

Differentiated thyroid cancer (DTC), arising from follicular epithelial cells, is the most prevalent type of thyroid malignancy. Papillary thyroid cancer (PTC) makes up approximately 85% of all DTC cases. According to the World Health Organization, PTCs with a maximum diameter ≤ 10 mm are categorized as subcentimeter PTCs (or papillary thyroid microcarcinomas (1-3).

The incidence of thyroid cancer has been increasing globally, mainly due to the overdiagnosis of this condition (4-6). The incidence of subcentimeter PTC has increased with the frequent use of sensitive imaging techniques, including ultrasonography, computed tomography, magnetic resonance imaging, and positron emission tomography. Additionally, exposure to radiation and carcinogenic substances has led to an increased incidence of thyroid cancer, which is almost exclusively attributed to papillary tumors (4,7). About half of the increased incidence is attributed to subcentimeter PTCs (8).

In general, subcentimeter PTCs have a good prognosis and a low mortality risk (9). Studies show that active surveillance management is a safe and effective alternative for subcentimeter PTCs in appropriately selected patients (10,11).

The optimal treatment for subcentimeter PTC remains undefined due to controversy about surgical treatment and the role of radioiodine therapy in this setting. It is essential to identify clinical and pathological features that contribute to the aggressiveness of subcentimeter PTC, such as lymph node involvement or distant metastases (12,13). Some studies have reported that lymph node metastases are more common in subcentimeter PTCs, especially in those with size > 5 mm (14,15). Additionally, studies have shown that extrathyroidal extension is significantly more frequent in large subcentimeter PTCs (16,17).

The 2015 guidelines of the American Thyroid Association (ATA) recommend lobectomy as the preferred treatment for individuals with subcentimeter PTCs, but recommendations are lacking in regard to the removal of the contralateral lobe in situations such as lymph node metastasis or family history of thyroid cancer. The guidelines propose a less aggressive approach with active surveillance, including serial ultrasonographic exams for low-risk tumors without metastasis, local invasion, or cytologic signs of aggressive disease. Despite the guidelines recommendations, the diagnosis of aggressive subtypes of PTC using fine-needle aspiration before surgery is challenging in clinical practice, with most cases identified after surgery. Thus, surgery remains a crucial step in confirming the diagnosis and initial treatment of aggressive PTC subtypes. The ATA recommends lobectomy for small unifocal intrathyroidal tumors and total thyroidectomy with therapeutic neck dissection (if lymph nodes are involved) for treating these aggressive subtypes, with prophylactic central compartment neck dissection recommended for T3-T4 tumors (2).

Considering the above, the aim of this study was to evaluate the clinicopathological features and follow-up outcomes of subcentimeter PTCs. The study also sought to identify factors affecting disease prognosis, including tumor size (≤5 mm *versus* >5 mm). The information collected will contribute to evidence supporting further studies on the management of these tumors.

## SUBJECTS AND METHODS

We conducted this retrospective study to identify the factors affecting the aggressiveness and prognosis of subcentimeter PTCs. The study protocol was approved by the local ethics committee (reference number 2022/380).

The study included 255 patients aged ≥ 18 years who underwent thyroid surgery at Ondokuz Mayıs University, Faculty of Medicine (Samsun, Turkey) between June 2008 and December 2021. Each patient had a pathology report of subcentimeter PTC – characterized by a maximum tumor diameter ≤ 10 mm – and underwent regular follow-up monitoring for at least 36 months. Thus, the inclusion criteria were (A) a diagnosis of subcentimeter PTC confirmed by pathology report, (B) a tumor size ≤ 10 mm, and (C) age ≥ 18 years. The only exclusion criterion was the absence of pre- and post-diagnosis data concerning PTC (notably, this criterion did not lead to the exclusion of any patient). The tumors were classified according to the American Joint Committee on Cancer (AJCC)/Union for International Cancer Control (UICC)/2017 TNM Staging System (12).

Information about the patients’ age, sex, surgical procedure, pathology result, recurrence, lymph node and distant metastases, mortality, and follow-up were collected from medical records. Postoperative evaluations were done immediately after surgery, at 3 and 6 months postoperatively, and subsequently at 3-12 month intervals. In these evaluations, the following aspects were noted: presence of poor prognostic factors, thyroid function tests, and requirements for radioiodine and hormone replacement therapy. The assessments at each follow-up visit were performed according to recommendations in the ATA guidelines and included measurement of serum levels of thyroglobulin, thyroid-stimulating hormone (TSH), and antithyroglobulin antibodies, as well as neck ultrasonography in search of lymph node metastasis, thyroid tissue remnants, and emergence of new thyroid nodules.

The histopathology of the surgical specimen was analyzed by an experienced pathologist affiliated with the institution. Information retrieved from the pathology reports included the tumors’ histopathologic subtype, diameter, bilaterality, multifocality, location within the gland, presence of capsule, capsular invasion, parenchymal invasion, necrosis, mitosis, calcification, vascular invasion, lymphatic invasion, perineural invasion, extrathyroidal extension, surgical borders, and presence of lymph nodes. The description of some of these features was absent in several pathology reports. Although features not mentioned in pathology reports are assumed to be absent in clinical practice, we opted in the present study to categorize and report these instances as “not mentioned” to avoid potential bias. All histopathologic features were categorized according to the current World Health Organization classification (1,3) and College of American Pathologists criteria (18). Risk classification was categorized according to the ATA risk classification system (2).

In the case of multifocality, the largest tumor size was considered. Radioiodine therapy, delivered by the center’s Nuclear Medicine department, was administered following protocols based on the clinician’s experience and established guidelines.

Two separate analyses were conducted as part of the study. The first analysis compared the variables of interest between two groups of patients: those with *versus* those without lymph node metastases. The second analysis also compared the variables of interest between two groups of patients: those with tumor size ≤ 5 mm *versus* those with tumor size > 5 mm (but ≤ 10 mm). In both analyses, the groups were compared in terms of age at diagnosis, sex, surgical procedure, pathology results, recurrence, distant metastasis, lymph node metastasis, radioiodine therapy, presence of disease-related death, and follow-up duration. As previously indicated, the pathology reports were prepared by an experienced pathologist affiliated with the institution.

### Statistical analysis

The data were analyzed using the software Statistical Package for Social Sciences (SPSS), Version 25 (IBM Corp., Armonk, NY, USA). P values < 0.05 were considered significant. Descriptive statistics are shown as mean ± standard deviation and median (minimum-maximum) values for numerical variables and number of observations and percentages for nominal variables. The Kolmogorov-Smirnov and Shapiro-Wilk tests were employed to determine if the distribution of numerical variables conformed to normality. The significance of differences between groups for numerical variables was evaluated using the independent samples *t* test for normally distributed data and the Mann-Whitney U test for non-normally distributed data, respectively. Nominal variables were evaluated using the chi-square test, Fisher’s exact test, and Fisher-Freeman-Halton exact test. Logistic regression analysis was performed to determine the factors affecting lymph node metastases, and odds ratios (OR), 95% confidence intervals (95% CIs), and p values for each variable were reported. Receiver operating characteristic (ROC) curve analysis was performed to determine the tumor size cutoff value identifying lymph node metastases.

## RESULTS

The first analysis of the study comparing the variables of interest between patients with *versus* without lymph node metastases included 255 patients (209 [82%] women, male-to-female ratio 1:4.5). The mean age at diagnosis was 48.52 ± 10.97 years. Overall, 181 (71%) patients were younger than 55 years. A breakdown of the age distribution by sex indicated that 155 (74.2%) and 54 (25.8%) women were younger than 55 years and 55 or older, respectively. The corresponding distribution among men was 26 (56.5%) and 20 (43.5%) respectively.

The surgical procedures performed were lobectomy in 19 (7.5%) patients, subtotal thyroidectomy in 9 (3.5%) patients, and total thyroidectomy in 227 (89%) patients. All patients received levothyroxine replacement. Radioiodine therapy was administered to 89 (34.9%) patients.

The patients were followed up for a median of 4 years (3-12 years). Four patients presented recurrence to cervical lymph nodes, including one who initially underwent subtotal thyroidectomy and three who underwent total thyroidectomy with lymph node dissection (none of these patients received radioiodine therapy).

Lymph node metastases were detected by ultrasonography in three patients: one at 6 months after subtotal thyroidectomy, another at both 3 and 6 months following total thyroidectomy with lymph node dissection, and a third patient 2 years after total thyroidectomy with lymph node dissection. Apart from the latter, the other two patients exhibited increased serum thyroglobulin levels. After evaluation of these patients, one underwent total thyroidectomy with lymph node dissection, and the others underwent only lymph node dissection. All these patients subsequently received radioiodine therapy.

Overall, one patient developed lung metastasis from PTC, which was diagnosed during investigation of a lung tumor; regional lymph node metastasis was not present in this case. Four patients died during follow-up, but none of these deaths were attributed to the subcentimeter PTC.

The mean tumor size in the overall sample was 5 mm (0.1-10 mm). Of the total, 137 (53.7%) patients had multifocal disease, and 23 (9%) exhibited capsular invasion. Vascular invasion was present in 5 (2%) patients, lymphatic invasion in 14 (5.5%), and extrathyroidal invasion in 2 (0.8%). Lymph node metastases were detected in 24 (9.4%) patients. Table 1 summarizes the histopathologic features of the tumors.

**Table 1:** Histopathologic features of the tumors

Focality n – (%)		Tumor size – average (mm)	5 (0.1-10)
Unifocal	118 (46.3)		
Multifocal	137 (53.7)		
**Histopathologic subtypes – n (%)**		**Calcification – n (%)**	
Classic	99 (38.8)	Yes	32 (12.5)
Tall cell	30 (11.8)	No	222 (87.1)
Solid	1 (0.4)	Not mentioned	1 (0.4)
Hobnail	2 (0.8)		
Follicular	109 (42.7)		
Clear cell	11 (4.3)		
Unspecified	3 (1.2)		
**Vascular invasion – n (%)**		**Lymphatic invasion – n (%)**	
Yes	5 (2)	Yes	14 (5.5)
No	249 (97.6)	No	240 (94.1)
Not mentioned	1 (0.4)	Not mentioned	1 (0.4)
**Capsular invasion – n (%)**		**Perineural invasion – n (%)**	
Yes	23 (9)	Yes	2 (0.8)
No	231 (90.6)	No	252 (98.8)
Not mentioned	1 (0.4)	Not mentioned	1 (0.4)
**Parenchymal invasion – n (%)**		**Extrathyroidal extension – n (%)**	
No	61 (23.9)	Yes	2 (0.8)
Yes	193 (75.7)	No	251 (98.4)
Not mentioned	1 (0.4)	Not mentioned	2 (0.8)
**Necrosis – n (%)**		**Surgical margin – n (%)**	
No	254 (99.6)	Intact	228 (89.4)
Not mentioned	1 (0.4)	Positive	24 (9.4)
		Not mentioned	3 (1.2)
**Mitosis – n (%)**		**Metastatic lymph nodes – n (%)**	
Yes	6 (2.4)	Yes	
No	248 (97.3)	No	24 (9.4)
Not mentioned	1 (0.4)		231 (90.6)

Abbreviation: n, number.

Patients with lymph node metastases (n = 24), compared with those without this complication (n = 231), were significantly younger at diagnosis and had significantly higher rates of recurrence and radioiodine therapy. Notably, lymph node metastases were more frequent in men (Table 2).

**Table 2:** Comparison of demographic data and general characteristics of patients categorized according to the presence of cervical lymph node metastases

	Lymph node metastasis		P values

	Present (n = 24)	Absent (n = 231)	
Age at diagnosis – years (mean ± SD)	42.96 ± 11.07	49.09 ± 10.82	0.009
Age groups – n (%)			
<55 years	21 (11.6)	159 (88.3)	0.094
≥55 years	3 (4.1)	72 (96)	
Sex – n (%)			
Female	15 (7.2)	194 (92.8)	0.021
Male	9 (19.6)	37 (80.4)	
Type of surgery – n (%)			
Lobectomy	0 (0)	19 (100)	0.387
Total thyroidectomy	23 (10.1)	204 (89.9)	
Subtotal thyroidectomy	1 (11.1)	8 (88.9)	
RAI treatment – n (%)			
Yes	23 (25.8)	66 (74.2)	< 0.001
No	1 (0.6)	165 (99.4)	
Recurrence – n (%)			
Yes	2 (50)	2 (50)	0.045
No	22 (8.8)	229 (91.2)	
Distant metastases – n (%) Pulmonary None			
Yes	0 (0)	1 (100)	1
No	24 (9.4)	230 (90.6)	
Death – n (%)			
Yes	0 (0)	4 (100)	1
No	24 (9.6)	227 (90.4)	
Suppressive therapy duration – years (mean ± SD)	2 (0-6)	2 (0-9)	0.271
Follow-up duration – years (mean ± SD)	3 (3-8)	4 (3-12)	0.165

Abbreviations: n, number; RAI, radioiodine; SD, standard deviation.

A comparison of pathology results between patients with *versus* without lymph node metastases showed that lymphatic invasion and calcifications (present in the tall-cell variant) were significantly more frequent in patients with lymph node metastases. Additionally, the tumor size in the group with lymph node metastases was significantly larger (Table 3).

**Table 3:** Histopathologic classification of the tumors categorized according to the presence or absence of cervical lymph node metastasis

	Lymph node metastases			P values

	Present (n = 24)		Absent (n = 231)	
Histopathologic subtypes – n (%)				
Classic	9 (9.1)	90 (90.9)	<0.001	
Tall cell	9 (30)	21 (70)		
Solid	0 (0)	1 (100)		
Hobnail	2 (100)	0 (0)		
Follicular	3 (2.8)	106 (97.2)		
Clear cell	1 (9.1)	10 (90.9)		
Unspecified	0 (0)	3 (100)		
Focality – n (%)				
Unifocal	8 (6.8)	110 (93.2)	0.262	
Multifocal	16 (11.7)	121 (88.3)		
Tumor size – mm	8 (2-10)	5 (0.1-10)	<0.001	
Capsular invasion – n (%)				
Yes	3 (13)	20 (87)	0.515	
No	21 (9.1)	210 (90.9)		
Not mentioned	0 (0)	1 (100)		
Parenchymal invasion – n (%)			0.134	
No	10 (16.4)	51 (83.6)		
Yes	14 (7.3)	179 (92.7)		
Not mentioned	0 (0)	1 (100)		
Necrosis – n (%)				
No	24 (9.4)	230 (90.6)	1	
Not mentioned	0 (0)	1 (100)		
Mitosis – n (%)				
Yes	1 (16.7)	5 (83.3)	0.504	
No	23 (9.3)	225 (90.7)		
Not mentioned	0 (0)	1 (100)		
Calcification – n (%)				
Yes	9 (28.1)	23 (71.9)	0.001	
No	15 (6.8)	207 (93.2)		
Not mentioned	0 (0)	1 (100)		
Vascular invasion – n (%)				
Yes	2 (40)	3 (60)	0.159	
No	22 (8.8)	227 (91.2)		
Not mentioned	0 (0)0	1 (100)		
Lymphatic invasion – n (%)				
Yes	8 (57.1)	6 (42.9)	<0.001	
No	16 (6.7)	224 (93.3)		
Not mentioned	0 (0)	1 (100)		
Perineural invasion – n (%)				
Yes	0 (0)	2 (100)	1	
No	24 (9.5)	228 (90.5)		
Not mentioned	0 (0)	1 (100)		
Extrathyroidal extension – n (%)				
Yes	0 (0)	2 (100)	1	
No	24 (9.6)	227 (90.4)		
Not mentioned	0 (0)	2 (100)		
Surgical margin – n (%)				
Intact	19 (8.3)	209 (91.7)	0.129	
Positive	5 (20.8)	19 (79.2)		
Not mentioned	0 (0)	3 (100)		

Abbreviation: n, number.

On multivariate logistic regression analysis, tumor size (OR 1.380, 95% CI 1.106-1.722, p = 0.004) and sex (OR 4.233, 95% CI 1.355-13.226, p = 0.013) were the main predictors of lymph node metastases (Table 4). Notably, lymph node metastases were more common in men than women and increased in frequency with the increase in tumor size.

**Table 4:** Multivariate logistic regression analysis of factors identifying the presence of lymph node metastases

	B	Sig	Exp(B)	95% CI for Exp(B)	

				Lower	Upper
Age at diagnosis	-.047	.071	.954	.907	1.004
Tumor size	.322	.004	1.380	1.106	1.722
Male sex	1.443	.013	4.233	1.355	13.226
Histopathologic subtype		.344			
Tall cell	1.213	.052	3.363	.989	11.436
Solid	-21.363	1.000	.000	.000	.
Hobnail	22.102	.999	396.4	.000	.
Follicular	-.768	.295	.464	.110	1.951
Clear cell	.209	.858	1.233	.124	12.218
Unspecified	-18.667	.999	.000	.000	.
Calcification		.806			
No calcification	.503	.511	1.654	.368	7.428
Not mentioned	-18.555	1.000	.000	.000	.
Lymphatic invasion		.054			
No lymphatic invasion	-1.668	.054	.189	.035	1.026
Constant	-1.586	.281	.205		

The following variables were used as references: female sex, classic tumor subtype, presence of calcification, presence of lymphatic invasion. Abbreviations: B, regression coefficient; Sig, significance value; Exp(B), odds ratio; 95% CI for Exp(B), 95% confidence interval of the odds ratio.

**Figure 1:**
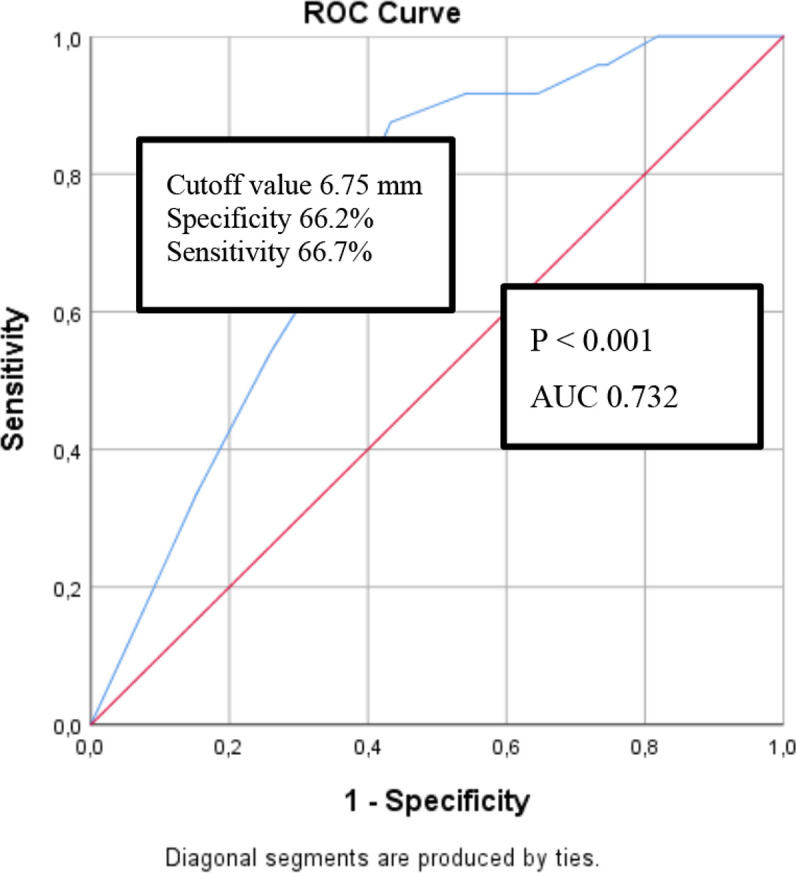
Receiver operating characteristic (ROC) curve analysis showing the tumor size cutoff value identifying the presence of lymph node metastases.

Abbreviations: AUC, area under the curve; ROC, receiver operating characteristic.

On ROC curve analysis, the tumor size cutoff value of 6.75 mm identified the presence of lymph node metastases with 66.2% specificity and 66.7% sensitivity (Figure 1).

The second analysis of the study comparing the variables of interest between patients grouped by tumor size included 246 patients. These patients were divided into two groups: those with tumor sizes ≤ 5 mm (n = 131, 53.3%) and those with tumor sizes > 5 mm group (n = 115, 46.7%).

In the ≤ 5 mm group, 59 (45%) tumors were multifocal, 7 (5.3%) had capsular invasion, 23 (17.6%) had parenchymal invasion, 8 (6.1%) had calcifications, 1 (0.8%) had vascular invasion, 1 (0.8%) had lymphatic invasion, 1 (0.8%) had extrathyroidal extension, 3 (2.3%) had lymph node metastases, and 31 (23.7%) were treated with radioiodine ablation therapy. In the > 5 mm group, 71 (61.7%) tumors were multifocal, 14 (12.2%) had capsular invasion, 37 (32.2%) had parenchymal invasion, 24 (20.9%) had calcifications, 3 (2.6%) had vascular invasion, 12 (10.4%) had lymphatic invasion, 1 (0.9%) had extrathyroidal extension, 21 (18.3%) had lymph node metastases, and 53 (46.1%) were treated with radioiodine ablation therapy. When the tumor size groups were compared in terms of pathology results, the > 5 mm group, compared with the ≤ 5 mm group, had higher rates of multifocality (p = 0.009), parenchymal invasion (p = 0.008), calcifications (p = 0.001), lymphatic invasion (p = 0.002), and lymph node metastases (p < 0.001). Additionally, radioiodine ablation therapy was administered more frequently to patients in the > 5 mm group (p < 0.001).

No significant differences were observed between the two tumor size groups in terms of follow-up duration (p = 0.234) or tumor recurrence (p = 1). The mean follow-up duration was 48 months (36-144) in both groups. Recurrence was detected in 2 (1.5%) patients with tumor size ≤ 5 mm and in 2 (1.7%) of those with tumor size > 5 mm. None of the patients in any of the groups experienced distant metastases or disease-related death. When the two groups were compared in terms of risk classification, 12 (9.2%) patients with tumor sizes ≤ 5 mm and 33 (28.7%) patients with tumor sizes > 5 mm were categorized in the intermediate risk group. In terms of surgery type (lobectomy, subtotal, and total thyroidectomy), no significant difference was observed between the ≤ 5 mm and > 5 mm groups (p = 0.633).

A significant difference between the ≤ 5 mm and > 5 mm groups was observed in terms of rates of histopathologic subtypes (p = 0.049), with the tall-cell variant being more common in the > 5 mm group (Table 5).

**Table 5:** Relationship between tumor size groups and tumor histopathologic subtype

Histopathologic subtypes	Tumor size group – n (%)		P value

	≤5 mm	>5 mm	
Classic	53 (55.2%)	43 (44.8%)	0.049
Tall cell	10 (33.3%)	20 (66.7%)	
Solid	0 (0%)	1 (100%)	
Hobnail	0 (0%)	2 (100%)	
Follicular	63 (59.4%)	43 (40.6%)	
Clear cell	5 (45.5%)	6 (54.5%)	

## DISCUSSION

A recent meta-analysis reported that the prevalence rates of small-sized PTCs in autopsy series are 14% in women and 11% in men (19). This suggests that subcentimeter PTCs can remain in a preclinical stage for years or decades. Although the clinical outcomes of subcentimeter PTCs are almost always excellent, regional recurrence, distant metastasis, and death can occur, albeit rarely (6). Some studies have observed no mortality from subcentimeter PTCs (10,14,20,21), while others have reported low mortality rates (0.3%-1%) associated with this type of tumor (2,22). In the present study, no disease-related mortality was observed among patients with subcentimeter PTCs over a median follow-up of 4 years.

Distant metastases at diagnosis are rarely seen in patients with subcentimeter PTCs. A meta-analysis including 9,313 patients with this type of tumor found that only 35 (0.37%) patients had distant metastases at diagnosis (22). In our sample, only 1 (0.4%) patient had distant metastasis present at the time of diagnosis.

In another recent meta-analysis, male sex, multifocality, tumor size > 5 mm, and extrathyroidal extension were found to be reliable clinical predictors of central lymph node metastasis in patients with clinically node-negative (CN0) subcentimeter PTCs (13).

Lymph node involvement is relatively common (12.3%-50%) in patients with subcentimeter PTC, and the incidence of this finding depends on the intensity of the investigation (20). Involvement of the central and ipsilateral lateral compartments has been observed in, respectively, 64% and 45% of the patients with subcentimeter PTC undergoing elective lymph node dissection (23). In our study, metastatic lymph nodes were detected in only 9.4% of the patients in the preoperative evaluation or in neck dissection performed due to clinical suspicion of lymph node metastases.

The clinical significance of calcification in thyroid malignancy, including subcentimeter PTCs, has not been established. In a study of patients with subcentimeter PTCs, those with calcifications had larger tumor sizes and more frequent and greater number of lymph node metastases than those without calcifications (24). Another study identified tumor calcification as an independent risk factor for central lymph node metastasis (25,26). Still, data on both calcifications and lymphatic invasions are scarce in studies comparing patients with subcentimeter PTCs ≤ 5 mm and > 5 mm. In our study, calcification and lymphatic invasion were significantly more frequent in patients with tumor size > 5 mm.

A positive correlation is generally observed between central lymph node metastases and multifocality in PTCs (1,27). Although studies analyzing both these complications in groups with subcentimeter PTCs ≤ 5 mm *versus* > 5 mm have found no significant correlation (16,28,29), some studies have reported more frequent multifocality in patients with tumors > 5 mm (30,31). In the present study, multifocality was significantly less frequent in the ≤ 5 mm group compared with the > 5 mm group.

In 1987, Kasai and cols. categorized subcentimeter PTCs according to size (≤5 mm *versus* > 5 mm) and found that the frequency of lymph node metastases and extrathyroidal invasion were higher in patients with tumor size > 5 mm, recommending more caution in the follow-up and treatment in these cases (31). Most studies adopt a cutoff value of 0.5 cm for tumor size when defining subcentimeter PTCs, and a tumor diameter > 0.5 cm has been identified as an associated risk factor for central lymph node metastases. While some studies have found nonsignificant differences in rates of lymph node metastases between ≤ 5 mm and > 5 mm tumors (15,19), others have reported significantly lower rates of this complication in tumors ≤ 5 mm (32-37).

Regarding subcentimeter PTC subtypes, studies have highlighted the presence of aggressive clinicopathological features in the tall-cell and hobnail variants (1,23,24,38). Additionally, a meta-analysis reported poor prognosis associated with the tall-cell variant of PTC, urging aggressive treatment in these cases (19). Another study found that angiolymphatic invasion, parenchymal invasion, extrathyroidal extension, and lymph node metastases were significantly more common in patients with the tall-cell compared with the classic variant, with the tall-cell variant emerging as an independent factor for poor prognosis compared with traditional variants (39). In a study adopting a cutoff value of 6 mm for PTC size, the tall-cell variant was significantly more frequent in the ≥ 6 mm group (40). Aligned with these findings, the tall-cell variant in the present study was significantly more frequent in the > 5 mm group. Additionally, parenchymal invasion was also significantly more frequent in this group.

Therapeutic central compartment lymph node dissection is performed routinely in patients with PTC associated with clinically significant lymph node metastases (2,20). In contrast, prophylactic central compartment lymph node dissection remains controversial, especially in patients with clinically node-negative subcentimeter PTCs, in whom personalized decision-making is recommended (2,20). While aggressive treatment often increases the risk of complications, such as hypoparathyroidism and recurrent laryngeal nerve injury, conservative treatment may increase recurrence rates (25). Our study found that male sex and large tumor size (cutoff value of 6.75 mm) increased the risk of cervical lymph node metastases. These factors should be considered during decisions about prophylactic central compartment neck dissection in patients with clinically node-negative subcentimeter PTCs.

The ATA guidelines recommend radioiodine therapy for patients with DTC categorized as intermediate-to-high risk (41). A meta-analysis has shown that radioiodine therapy of PTC improves the patients’ prognoses by increasing the rates of long-term disease-free survival and decreasing the rates of regional recurrence, distant metastases, and mortality (42). A study analyzing patients with PTC found a disease-related mortality rate of 0.3%, along with recurrence rates of 6% and 8% at 20 years and 40 years, respectively (14). In the present study, 2 (1.5%) patients with tumor size ≤ 5 mm and 2 (1.7%) of those with tumor size > 5 mm presented tumor recurrence. The low recurrence rates associated with ≤ 5 mm and > 5 mm tumors may be related to the short follow-up period. None of the patients experienced disease-related mortality.

The limitations of the present study are its single-center, retrospective design and the inclusion of a small sample size. Additionally, the follow-up period was relatively short (median 4 years), which may not have been sufficient for a comprehensive assessment of recurrence rates. As an important strength of our study, the histopathologic analyses were reviewed by an experienced pathologist specialized in endocrine pathology. Multicenter, prospective studies with a greater number of cases and comprehensive analysis of molecular markers should be conducted to identify more clearly the factors contributing to aggressiveness in subcentimeter PTCs.

In conclusion, the present retrospective study analyzed several factors associated with prognosis and treatment outcomes in subcentimeter PTCs. These tumors can remain in a preclinical stage for years or decades. While their clinical outcomes are generally excellent, regional recurrence, distant metastasis, and death may rarely occur. The study results indicated that male sex and tumor size > 6.75 mm were reliable clinical predictors of central lymph node metastasis in clinically node-negative subcentimeter PTC. Age, histopathologic subtype, presence of calcification, and lymphatic invasion emerged as significant factors influencing lymph node metastasis in the univariate analysis but did not remain as significant independent predictors in the multivariate logistic regression analysis. Distant metastasis is rarely observed in patients with subcentimeter PTC at the time of diagnosis. Lymph node involvement is relatively common in these tumors (12.3%-50%, depending on the extent of the investigation). Additionally, the tall-cell variant and tumor calcification were significant predictive factors of lymph node metastases in the univariate analysis, but this association lost significance in the multivariate regression analysis. Decisions for prophylactic central compartment neck dissection should take into account risk factors such as male sex and tumor size. Radioiodine therapy is recommended for patients with DTC classified as intermediate or high risk. The present study also observed similar recurrence rates in patients with tumors ≤ 5 mm *versus* > 5 mm and no disease-related mortality. Since recurrence was seen in patients who did not undergo radioiodine therapy, we hypothesize that this therapy would probably have decreased the risk of recurrence, although no significant difference (p = 0.305) was observed in terms of risk of recurrence between patients who did *versus* did not undergo radioiodine therapy, probably due to the small number of patients who experienced recurrence. Finally, predictive factors associated with tumor aggressiveness were significantly more frequent in patients with tumors > 5 mm. We recommend that patients with subcentimeter PTCs > 5 mm who present with the predictive factors identified in this study be managed and followed up more carefully. Future studies should be conducted to evaluate the treatment modalities and recurrence patterns in patients with subcentimeter PTC.
